# Comparing the novel microstream and the traditional mainstream method of end-tidal CO_2_ monitoring with respect to PaCO_2_ as gold standard in intubated critically ill children

**DOI:** 10.1038/s41598-020-79054-y

**Published:** 2020-12-16

**Authors:** Muhterem Duyu, Anıl Dogan Bektas, Zeynep Karakaya, Meral Bahar, Aybuke Gunalp, Yasemin Mocan Caglar, Meryem Nihal Yersel, Ozlem Bozkurt

**Affiliations:** 1 Department of Pediatrics, Pediatric Intensive Care Unit, Istanbul Medeniyet University Goztepe Training and Research Hospital, Istanbul, Turkey; 2 Department of Pediatrics, Istanbul Medeniyet University Goztepe Training and Research Hospital, Istanbul, Turkey

**Keywords:** Paediatrics, Paediatric research, Medical research

## Abstract

The objective of this study was to evaluate a novel microstream method by comparison with PaCO_2_ and the more standard mainstream capnometer in intubated pediatric patients. We hypothesized that the novel microstream method would superior compared to the traditional mainstream method in predicting PaCO_2_. This was a prospective single-center comparative study. The study was carried out on 174 subjects with a total of 1338 values for each method. Data were collected prospectively from mainstream and microstream capnometer simultaneously and compared with PaCO_2_ results. Although both mainstream PetCO_2_ (mainPetCO_2_) and microstream PetCO_2_ (microPetCO_2_) were moderately correlated (r = 0.63 and r = 0.68, respectively) with PaCO_2_ values, mainPetCO_2_ was in better agreement with PaCO_2_ in all subjects (bias ± precision values of 3.8 ± 8.9 and 7.3 ± 8.2 mmHg, respectively). In those with severe pulmonary disease, the mainPetCO_2_ and microPetCO_2_ methods were highly correlated with PaCO_2_ (r = 0.80 and r = 0.81, respectively); however, the biases of both methods increased (14.8 ± 9.1 mmHg and 16.2 ± 9.0 mmHg, respectively). In cases with increased physiologic dead space ventilation, the agreement levels of mainPetCO_2_ and microPetCO_2_ methods became distorted (bias ± precision values of 20.9 ± 11.2 and 25.0 ± 11.8 mm Hg, respectively) even though mainPetCO_2_ and microPetCO_2_ were highly correlated (r = 0.78 and r = 0.78, respectively). It was found that the novel microstream capnometer method for PetCO_2_ measurements provided no superiority to the traditional mainstream method. Both capnometer methods may be useful in predicting the trend of PaCO_2_ due to significant correlations with the gold standard measurement in cases with severe pulmonary disease or increased physiological dead space –despite reduced accuracy.

## Introduction

The monitoring of carbon dioxide (CO_2_) level is essential for diagnosis and therapeutic guidance in mechanically ventilated patients^1^. The current gold standard method for the measurement of partial pressure of carbon dioxide in the blood (PaCO_2_) is the arterial blood gas (ABG) method. But ABG is an invasive method and does not provide continuous monitoring^2^.

Capnometers, which continuously monitor PCO_2_ levels and display the waveform of PCO_2_ in exhaled air non-invasively, provide information on the adequacy of ventilation^3^. Detection of exhaled PCO_2_ (end-tidal PCO_2_) has proven to be a valuable mechanism to confirm tracheal intubation and to recognize accidental esophageal intubations, among other critical patient safety benefits^4^. The patient protection enhancements provided by end-tidal PCO_2_ (PetCO_2_) monitoring also include the detection of invasive airway disconnection, dislodgement or obstruction, prediction of underlying airway or lung pathologies and monitoring the effectiveness of cardiopulmonary resuscitation^3,5,6^.

It is possible to measure PetCO_2_ by mainstream or sidestream capnometer technologies. The technique is named based on the localization of the PetCO_2_ sensor^7,8^. Mainstream capnometers are devices in which the infrared source and PCO_2_ detector are placed between the proximal endotracheal tube (ETT) and the ventilator circuit^7,9^. On the other hand, sidestream capnometers aspirate samples from the airway through tubing attached to a sampling line and airway adapter between the ETT and the ventilation circuit. Sidestream methods utilize an infrared PCO_2_ sensor in a monitor that may be located far from the patients^10^. A new technology for sidestream systems (Microstream, Oridion Medical, Inc., Danville, CA) is now available that uses very low flow rates (50 mL/min) to preserve the accuracy and resolution of the waveform as well as eliminating secretion/moisture-related occlusion problems by the use of special filters^7^.

There are many studies evaluating the accuracy of mainstream, sidestream and microstream capnometer technologies in the literature^11–15^. Critics of capnometer usage cite multiple studies which demonstrate that PetCO_2_ and PaCO_2_ do not reliably correlate in some clinical situations^12,16,17^. The analyses utilized in these studies are highly variable and fail to consider physiologic dead space/severity of pulmonary disease and/or their effect on the relationship between PaCO_2_ and PetCO_2_. Additionally, there are few studies that have compared different PetCO_2_ monitoring techniques with each other^18–21^.

This study was undertaken to evaluate the correlations of gold standard PaCO_2_ measurements with the microstream and mainstream PetCO_2_ capnometers, and to compare the accuracy and results of the latter two methods among ventilated patients in the pediatric intensive care unit (PICU). We hypothesize that, (i) in intubated pediatric patients, the microstream technology will allow better prediction of PaCO_2_ compared to the traditional mainstream method, and that (ii) microstream measurements predict PaCO_2_ more reliably than mainstream measurements across increased levels of dead space ventilation and in the presence of severe pulmonary disease—after controlling for the expected PetCO_2_-PaCO_2_ gradient.

## Methods

This prospective, single-center comparative study was conducted at the PICU of Medeniyet University, Goztepe Training and Research Hospital (Istanbul, Turkey) between January 2018 and July 2019. All procedures and processes were carried out according to principles mentioned in the Helsinki Declaration and the Good Clinical Practice guideline.

### Population

The study evaluated all children aged between 1 month to 17 years that had been intubated with cuffed ETT due to a definite indication for mechanical ventilation. Among these, those who accepted invasive monitoring of arterial blood pressure and provided informed consent (parents or legal guardians) were included in the study. The presence of any one of the following characteristics was defined as grounds for exclusion from the study: patients with tracheostomy, sampling performed with venous blood, non-compliance to study protocol (premature discontinuation of measurement, signal abnormality [absence of waveform or presence of interrupted waveform]), use of uncuffed endotracheal tubes, patients with known congenital heart and lung defects, need for high-frequency oscillatory ventilation or extracorporeal life support, determination of any type of air leakage in the lung (pneumothorax, pneumomediastinum etc.).

### Monitoring

The intubations were performed with single-lumen cuffed ETT that was appropriately sized for age and weight. CO_2_ in the exhaled air of patients was monitored simultaneously with mainstream (Mainstream EtCO_2_; Philips Capnostat M25O1A, Germany) and microstream (Microstream EtCO_2_; Medtronic Capnostream35, USA) capnometers. The dimensions of the airway adapters to be used were based on the manufacturer's guidelines. The airway adaptors of both methods of measurements were kept in the same location and insertions were performed sequentially between the airway circuit and the proximal ETT. ABG were analyzed at the bedside using an ABL 90 FLEX blood gas analyzer (Radiometer, Medical ApS, Copenhagen, Denmark) within 3 min of collection and without any delay. No additional ABG was performed for the data collection of consecutive samples.

### Study protocol and recording

ABG analysis, mainPetCO_2_ and microPetCO_2_ values and mechanical ventilator parameters were recorded simultaneously. Prior to obtaining each arterial blood gas sample, a researcher checked whether the capnometer adapters were blocked by secretions or moisture. Capnometer adapters were replaced with new ones in the event of any type of blockage. Both capnometer methods were analyzed using continuous steady waveforms of expired CO_2_ through the ventilator cycle, in order to ensure the accuracy of readings. A minimum of 4 and a maximum of 8 simultaneous PCO_2_ measurements (PaCO_2_, mainPetCO_2_, microPetCO_2_) were planned to be taken from each patient. Patients with a measurement number less than 4 for various reasons (death, extubation, interruption of monitoring etc.) were excluded from the study.

Patients’ demographic characteristics and their clinical and laboratory parameters were identified (gender, age [months] and primary diagnosis). The parameters of mechanical ventilation, including FiO_2_ (Fractional inspired oxygen), mean airway pressure (MAP) were recorded in addition to PetCO_2_ values (mainPetCO_2_ and microPetCO_2_), parameters of arterial blood gas analysis (pH, PaCO_2_, PaO_2_, HCO_3_ˉ) and oxygenation index (OI) (OI = [FiO_2_ × MAP × 100)/PaO_2_])^22^. Lung physiologic dead space volume is defined as wasted tidal volume during respiration (i.e., the volume remaining in the conducting airways [anatomical dead space] and in poorly perfused and non-perfused alveoli [alveolar dead space] that are not participating in gas exchange). A ratio of dead space volume to tidal volume (Vd/Vt) was calculated using the Enghoff modification of the Bohr equation: Vd/Vt = [PaCO_2_ − PetCO_2_]/PaCO_2_^23^. Dead space ventilation was calculated separately using PetCO_2_ values obtained from microstream and mainstream methods.

For subgroup analyses, patients were grouped with regard to the severity of pulmonary disease and physiological dead space ventilation levels. Severe pulmonary disease was defined as an OI of ≥ 10 and mild-to-moderate pulmonary disease was defined as an OI of < 10^18,24^. Determination of Vd/Vt ratio ≥ 0.4 was defined as increased dead space ventilation^23,25^. The consistency of PetCO_2_ monitoring (mainPetCO_2_ and microPetCO_2_) within each patient and subgroup was assessed by examining the relationships between the changes in PaCO_2_ and the two PetCO_2_ methods in consecutive samples.

### Statistical analysis

Analyses were performed by using the IBM Statistical Package for the Social Sciences version 21 (SPSS, Inc., Chicago, IL) or Med Calc version 19.1 (Med Calc Software, Ostend, Belgium). Patient characteristics are described using qualitative variables (using frequencies and percentages) and quantitative variables (using means and standard deviation [SD] or median with interquartile range [IQR], depending on type of distribution). Simple linear regression analysis was performed and Spearman correlation coefficients were calculated for the assessment of relationships between PaCO_2_, main-PetCO_2_ and micro-PetCO_2_. We assessed the agreement between these measurements (bias [mean difference] and precision [SD of the differences]) by the Bland–Altman technique. The results were considered statistically significant in tests resulting in a* P* value lower than 0.05.

### Power analysis

Power analysis was conducted using the Power Analysis Sample Size (PASS) for Windows version 11.0 Package Program. Group sample sizes of 174 were determined to achieve 97% power to detect a difference of − 3.6 between the null hypothesis that both group means were 3.8, and the alternative hypothesis that the mean of group 2 was 7.4 (with estimated group standard deviations of 9.0 and 8.3), and with a significance level (alpha) of 0.05 using a two-sided two-sample t-test.

### Ethics approval and consent to participate

The study was approved by the institutional review board of our center. The parents of all patients signed an informed consent form before inclusion into the study.

### Conference presentation

This study presented at the 15th National Congress of Pediatric Emergency and Critical Care, 18–20 October 2018, Turkey.

## Results

The study was performed in 174 patients that provided 1338 measurements for each method. The median age and interquartile range (IQR) of the included subjects was 42 months (IQR: 12–108 mo.). Table 1 shows the characteristics of the study group. Conventional invasive mechanical ventilator modes were used in all patients included in the study (Galileo Mechanical Ventilator, Hamilton Medical AG, Rhäzüns, Switzerland).

**Table 1:** Demographic, clinical and laboratory characteristics of patients (n = 174).

Patients characteristics	Values
Male sex, no (%)	107 (61.5)
Age (month), median (IQR)	42 (12–108)
**Primary disease, no (%)**	
Pneumonia	39 (22.4%)
Multiple trauma	29 (16.7%)
Status epilepticus	22 (12.6%)
Shock, multiple organ failure	19 (10.9%)
Postoperative	10 (5.8%)
Bronchiolitis	9 (5.2%)
Intracranial mass/hemorrhage	8 (4.6%)
Central nervous system infection	7 (4.0%)
Acute respiratory distress syndrome	5 (2.8%)
Congenital heart disease	5 (2.8%)
Poisoning	4 (2.3%)
Renal failure	4 (2.3%)
Others	13 (7.6%)
**Laboratory values, median (IQR)**	
Arterial blood gas analysis	
pH	7.3 (7.3–7.4)
PaCO_2_ (mm Hg)	40.7 (35.4–47.3)
PaO_2_ (mm Hg)	150.0 (115.0–183.0)
HCO_3_ˉ (mmol/L)	22.5 (19.7–25.3)
MainPetCO_2_, (mm Hg)	38.0 (32.0–44.0)
MicroPetCO_2_, (mm Hg)	35.0 (29.0–40.0)
**Mechanical ventilator parameters, median (IQR)**	
FiO_2_ (%)	40.0 (40.0–50.0)
Mean airway pressure (mm Hg)	10.0 (9.0–13.0)
Oxygenation Index	2.4 (1.6–3.9)

PaCO_2_ = arterial PCO_2_, PaO_2_ = arterial PO_2_, mainPetCO_2_ = mainstream end-tidal PCO_2_, microPetCO_2_ = microstream end-tidal PCO_2_, FiO_2_ = fractional inspired oxygen, oxygenation index = [(FiO_2_ × MAP × 100)/PaO_2_], IQR: Interquartile range.

**Figure 1:**
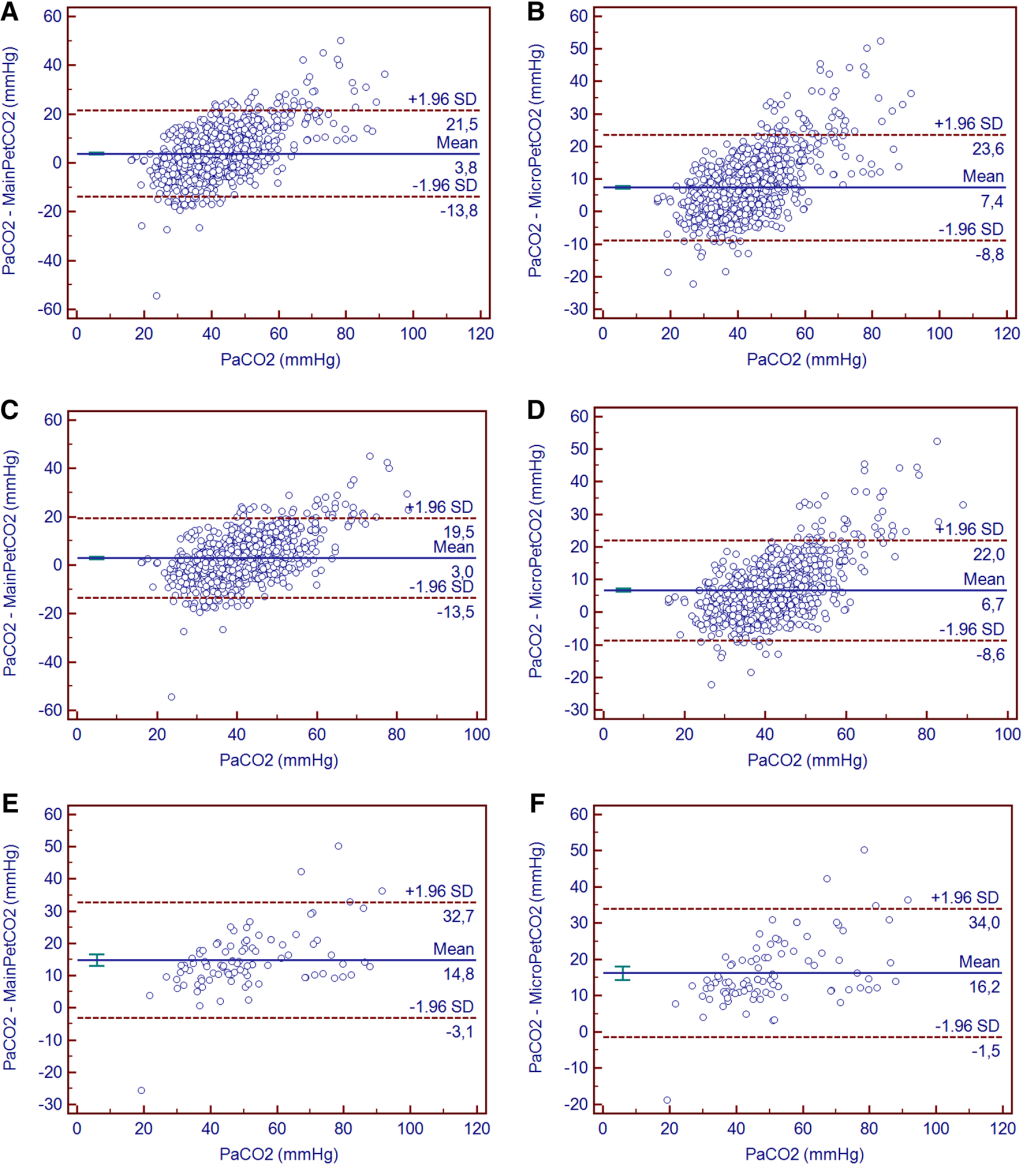
Bland–Altman plots for mean mainPetCO_2_ versus PaCO_2_ (**A**) and mean microPetCO_2_ versus PaCO_2_ (**B**) for all patients, mainPetCO_2_ and PaCO_2_ for the patients with mild to moderate pulmonary disease (**C**), microPetCO_2_ and PaCO_2_ for the patients with mild to moderate pulmonary disease (**D**), mainPetCO_2_ and PaCO_2_ for the patients with severe pulmonary disease (**E**), microPetCO_2_ and PaCO_2_ for the patients with severe pulmonary disease (**F**). The mean difference is represented as a continuous line, and 95% limits of agreement are represented as dotted lines.

**Figure 2:**
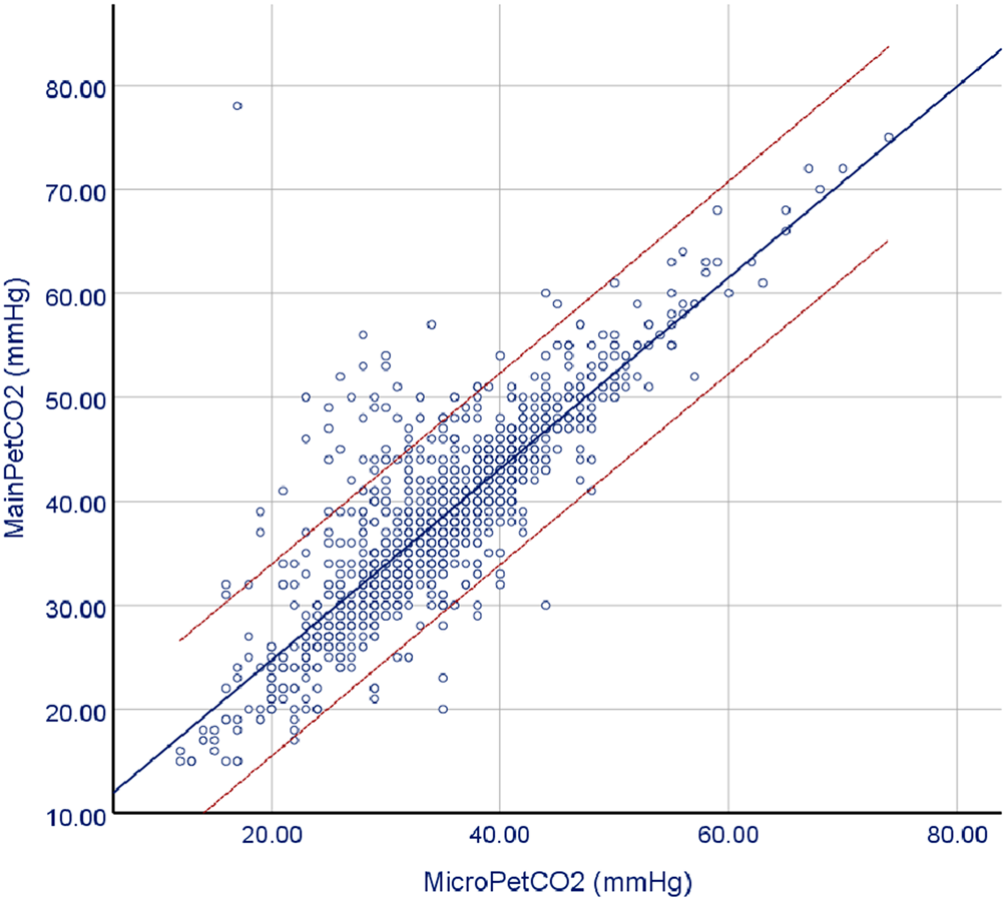
The relationship between mainPetCO_2_ and microPetCO_2_ measurements for all subjects.

The median (range) levels of PaCO_2_, mainPetCO_2_, and microPetCO_2_ were 40.7 (IQR: 35.4–47.3) mm Hg, 38.0 (IQR: 32.0–44.0) mm Hg, and 35.0 (IQR: 29.0–40.0) mm Hg, respectively. The results of the Bland–Altman analysis comparing mainPetCO_2_/PaCO_2_ and microPetCO_2_/PaCO_2_ pairs are summarized in Table 2 and illustrated in Fig. 1 in all subject groups—and also according to severity of pulmonary disease. In all subjects (1338 pairs), the mean difference (bias) and SD of the differences (precision) for mainPetCO_2_ was 3.8 ± 8.9 mm Hg (95% limits of agreement -13.7 to 21.4 mm Hg) with moderate correlation (r = 0.63, *p* < 0.001) (Fig. 1A). The mean bias and precision for microPetCO_2_ was 7.3 ± 8.2 mm Hg (95% limits of agreement − 8.8 to 23.6 mm Hg) with moderate correlation (r = 0.68, *p* < 0.001) (Fig. 1B). Although both PetCO_2_ measurement methods were moderately correlated, mainPetCO_2_ was more accurate compared to the microPetCO_2_ method overall (in the whole subject group)_._ Additionally, when we evaluated the correlation between mainPetCO_2_ and microPetCO_2_ throughout all patients, the methods demonstrated a strong level of correlation (r = 0.84, *p* < 0.001) (Fig. 2).

**Table 2:** Relation between PetCO_2_ values and severity of pulmonary disease.

Parameter	Mean difference ± SD (mmHg)	95% LLA (mmHg)	95% ULA (mmHg)	r	*p*
**In All Subjects (n = 1338 pairs)**					
PaCO_2_–MainPetCO_2_	3.83 ± 8.99	− 13.79	21.46	0.63	< 0.001
PaCO_2_–MicroPetCO_2_	7.39 ± 8.27	− 8.83	23.61	0.68	< 0.001
**Severity of pulmonary disease**					
**Mild to moderate pulmonary disease**^**a**^					
PaCO_2_–MainPetCO_2_	2.98 ± 8.40	− 13.49	19.46	0.64	< 0.001
PaCO_2_–MicroPetCO_2_	6.70 ± 7.80	− 8.59	22.01	0.68	< 0.001
**Severe pulmonary disease**^**b**^					
PaCO_2_–MainPetCO_2_	14.83 ± 9.12	− 3.06	32.72	0.80	< 0.001
PaCO_2_–MicroPetCO_2_	16.24 ± 9.05	− 1.49	33.99	0.81	< 0.001

All CO_2_ levels in mmHg. LLA = lower limit of agreement, ULA = upper limit of agreement, SD = standard deviation, PCO_2_ = partial pressure of carbon dioxide, PaCO_2_ = arterial PCO_2_, mainPetCO_2_ = mainstream end-tidal PCO_2_, microPetCO_2_ = microstream PCO_2_.

^a^Definition of mild to moderate pulmonary disease: oxygenation index < 10 (n = 1242 pairs).

^b^Definition of severe pulmonary disease: oxygenation index ≥ 10 (n = 96 pairs).

Study subjects were also compared based on the presence of lung pathology (Table 2). In the mild-to-moderate pulmonary disease group, 1242 measurements from each end-tidal CO_2_ method were compared. In this group, the mean bias and precision for mainPetCO_2_ was 2.9 ± 8.4 mm Hg (95% limits of agreement − 13.4 to 19.4 mm Hg) with moderate correlation (r = 0.64, *p* < 0.001) (Fig. 1C). The mean bias and precision for microPetCO_2_ was 6.7 ± 7.8 mm Hg (95% limits of agreement − 8.5 to 22.1 mm Hg) with moderate correlation (r = 0.68, *p* < 0.001) (Fig. 1D). Although both PetCO_2_ measurement methods were moderately correlated, mainPetCO_2_ was more accurate than microPetCO_2._

In the severe lung disease group, we compared 96 results from each method of measurement. For the mainPetCO_2_ and PaCO_2_ comparison, the mean bias and precision was 14.8 ± 9.1 (95% limits of agreement − 3.0 to 32.7 mm Hg) (Fig. 1E). Whereas the mean bias and precision between microPetCO_2_ and PaCO_2_ was 16.2 ± 9.0 mm Hg (95% limits of agreement − 1.4 to 33.9 mm Hg) (Fig. 1F). In the severe lung disease group, almost all PaCO_2_ values were higher than PetCO_2_ measurements (Fig. 1E,F). Additionally, in this group, both mainPetCO_2_ and microPetCO_2_ were highly correlated with PaCO_2_ (r = 0.80, *p* < 0.001 and r = 0.81, *p* < 0.001, respectively); however, the biases of both methods increased.

To determine whether the accuracy of the non-invasive PCO_2_ measurement methods were altered in the presence of high physiologic dead space, we compared the mainPetCO_2_ and microPetCO_2_ values with regard to groups formed according to Vd/Vt ratio (< 0.4 vs. ≥ 0.4). The Vd/Vt ratio was < to 0.4 in 1247 of 1338 (93%), and > 0.4 in 91 (7%) measurements.

In the Vd/Vt < 0.4 (the normal physiologic dead space) group, the comparison of mainPetCO_2_ and PaCO_2_ values showed a mean bias and precision of 3.0 ± 8.0 mm Hg, with moderate correlation (r = 0.63, *p* < 0.001). Whereas the mean bias and precision between microPetCO_2_ and PaCO_2_ was 6.5 ± 7.0 mm Hg, again with moderate correlation (r = 0.68, *p* < 0.001). In the Vd/Vt ≥ 0.4 (increased physiologic dead space) group, both mainPetCO_2_ and microPetCO_2_ were highly correlated (r = 0.78, *p* < 0.001 and r = 0.78, *p* < 0.001, respectively) with increased PetCO_2_—PaCO_2_ gradient (bias ± precision values of 20.9 ± 11.2 and 25.02 ± 11.8 mm Hg, respectively). Although both non-invasive PCO_2_ measurement methods were highly correlated with PaCO_2_, mainPetCO_2_ was more accurate than microPetCO_2_ in both the normal and increased dead space ventilation groups.

## Discussion

To our knowledge, this is the largest cohort study including 174 pediatric patients who received mechanical ventilation in the PICU. The evaluation of 1338 measurements for each method and the comparison of two different PetCO_2_ monitoring methods with accuracy determined according to simultaneous PaCO_2_ measurements are among the other strengths of this study. Although different PetCO_2_ measurement methods have distinct advantages, the accuracy and correlation of these methods in comparison to ABG measurements is without doubt the most vital feature of any method. The microstream capnometer requires in-depth analysis to prove that it contributes to or surpasses available methods by analyzing whether the advantageous properties expressed in the literature are indeed superior in the real-life follow-up of intubated pediatric patients.

Although there are many studies evaluating the accuracy and correlation of various non-invasive PetCO_2_ measurement methods, the majority of these studies were performed in non-intubated patient groups^26–30^. In intubated patients, the studies on PetCO_2_ monitoring are mostly compared with the ABG analysis of a single method and often evaluate the relationship between the severity of lung disease and the accuracy of the method. In our study, two different PetCO_2_ monitoring methods were evaluated simultaneously, and both mainPetCO_2_ and microPetCO_2_ measurements were found to be moderately correlated with PaCO_2_.

Rozycki et al. reported that mainPetCO_2_ measurements were highly correlated with PaCO_2_ in intubated newborns, with a mean bias of -6.9 mm Hg^13^. Similar results have been found in other studies using the mainstream technology in intubated newborns^31,32^. Microstream is preferred especially in the neonatal age group due to the use of very low flow rates (50 mL/min), causing smaller dead space and allowing measurement from the distal part of the ETT. In the study by Kugelman et al.^24^ microPetCO_2_ was found in adequate agreement with PaCO_2_, which indicated closer agreement than seen in the current research. Although similar ‘close’ results have been obtained in other studies^18,33^, Singh and colleagues found similar results to ours in terms of agreement between microPetCO_2_ and PaCO_2_^15^. In intubated patients, PetCO_2_ measurements can be performed from the proximal or distal part of the ETT. To compare the advantages of different PetCO_2_ measurement technologies in our study, it was thought that the measurements obtained from the same locations would be more guiding. Therefore, in order for one of the methods to gain no advantage due to localization, both PetCO_2_ measurements were obtained from the same location (proximal part of ETT). In various studies comparing PetCO_2_ measurements obtained from the distal and proximal parts of the ETT, it has been suggested that distal measurements provide more accurate results; however, several other studies have demonstrated comparable accuracy between proximal and distal measurements^21,33–35^.

The first study comparing two different PetCO_2_ measurement methods in intubated patients was performed by Kugelman and colleagues. This study, which was comprised of 27 infants, showed better correlation between PetCO_2_ and PaCO_2_ with distal sampling of expired air using microstream technology against the mainstream method through a proximal port using double lumen ETT^18^. The measurements made in this study were obtained from different locations of the ETT and this situation may have led to an advantage for the microstream method. In our study, although the correlation coefficients of both methods were similar, the agreement level of main PetCO_2_ measurements was better.

There are various studies investigating the relationships between pulmonary disease and PaCO_2_-PetCO_2_ values. These studies have defined pulmonary disease severity according to various parameters, such as OI, arterial-alveolar PO_2_ gradient and PaO_2_/FiO_2_ ratio. In this study we used the OI value to define severe pulmonary disease. Sivan et al. reported in their study that mainPetCO_2_ and PaCO_2_ compatibility decreased as lung disease severity increased in neonatal patients^36^. Hagerty et al. evaluated the compatibility of microPetCO_2_ and PaCO_2_ in intubated newborn patients and found that microPetCO_2_ and PaCO_2_ differences were higher in the pulmonary disease group compared to controls^33^. Different results were reported by other investigators. Tingay et al.^37^ found that the PetCO_2_ bias was independent of severity of lung disease and similarly Rozycki et al.^13^ reported that the degree of lung disease had little influence on the degree of discrepancy between measurement. Kugelman and colleagues reported that although the accuracy of microPetCO2 decreased with lung disease it still remained good correlation as a useful measure of PaCO2 in conditions of severe lung disease^18^. The study by McDonald et al. found an overall moderately correlation between PaCO_2_ and mainPetCO2 for all included patients, but the investigators concluded that significant lung disease (defined by PaO_2_/FiO_2_ < 200) had a negative effect on the correlation^12^. In our study, it was concluded that both PetCO_2_ measurement methods highly correlated in patients with severe lung disease, albeit with a significant decrease in measurement accuracy.

The most important parameter contributing to the PetCO_2_-PaCO_2_ gradient is the increase in physiological dead space due to ventilation-perfusion mismatch^38,39^. Physiologic dead space ventilation is the sum of anatomical dead space from the conducting airways and alveolar dead space from disease processes and/or therapies employed. The increased gradient between PetCO_2_ and PaCO_2_ with high PaCO_2_ levels are directly proportional to the degree of physiologic dead space. Although typical alveolar CO_2_ concentrations are slightly greater than of ABG, PetCO_2_ normally 2–5 mmHg lower than PaCO_2_ due to mixing of CO_2_ containing alveolar gas with exhaled gas devoid of CO_2_ from the anatomical dead space. In a patient with lung disease, the addition of alveolar dead space further dilutes PetCO_2_ relative to PaCO_2_. As a result, PetCO_2_ measurements depict greatly reduced results compared to PaCO_2_. The normal physiologic dead space to tidal volume ratio (Vd/Vt) is established to be 0.20–0.35^23^. In this study, we provide evidence that physiologic dead space ventilation is a major factor in determining the relationship between capnographic monitoring of PetCO_2_ and PaCO_2_. Despite multiple earlier publications comparing PetCO_2_ and PCO_2_ in presence of pulmonary disease and hypercarbia, few studies have examined the effect of change in physiologic dead space on the relationship between PetCO_2_ and PaCO_2_ across an increased range of Vd/Vt ratios in mechanically ventilated pediatric patients^23^. Our study is the first to investigate the correlations between two different capnometers in patients with increased physiological dead space ventilation.

In patients with a low calculated physiologic dead space to tidal volume ratio (Vd/Vt < 0.4), there is a moderate correlation between both PetCO_2_ (measured noninvasively by capnography) measurements and PaCO_2_ value. Despite the high correlation between PetCO_2_ and PaCO_2_ values in patients with high physiologic dead space to tidal volume ratio (Vd/Vt ≥ 0.4), the accuracy of measurements was greatly reduced. Therefore, in the presence of severe pulmonary diseases with increased physiological dead space, it is much more reliable to use PetCO_2_ results as a measure of trend rather than absolute value. It is also critical to note that further problems in accuracy may arise with smaller infants or newborns (which were not included in the study population) and reduced volumes or I:E values. In a study including 56 intubated pediatric patients by McSwain et al., it was found that, while the strength of the association diminished slightly as the dead space ratio increased, the correlation still remained strong between the methods. The PaCO_2_-PetCO_2_ gradient was increased predictably with increasing Vd/Vt^23^. Our findings show that increased physiological dead space as a result of severe pulmonary disease will increase the gradient between PaCO_2_ and PetCO_2_ in favor of PaCO_2_ values, making almost all PaCO2 results greater than those recorded by PetCO_2_. These findings were similar to the outcomes of previous studies performed in newborns and children with pulmonary disease^33,36^.

To our knowledge, there are no other studies investigating the relationships between PetCO_2_ measurements and increased physiological dead space ventilation. There are however, various studies investigating PetCO_2_ correlations with hypercarbia as a proxy for increased dead space ventilation. In the study conducted by Kugelman et al., microPetCO_2_ was reported as a useful measure of PaCO_2_, whereas mainPetCO_2_ was distorted on the high range of PaCO_2_ level^18^. Rosycki et al.^13^ did not find any effect of increased PaCO_2_ on mainPetCO_2_ measurements.

Our study has several limitations. Non-consecutive ABGs were used for data collection and inadvertent selection bias may have been introduced. In our study, we used proximal measurement method for both PetCO_2_ methods. In subsequent studies, the relationship between concurrent microPetCO_2_ measurements obtained from the proximal and distal part of the ETT may reveal differences in results which could be crucial for physicians and patients in intensive care units. Although our study reached the highest number of patients and samples in the literature, the number of samples in the subgroups of severe pulmonary disease and increased physiologic dead space ventilation, were relatively low; thus limiting the generalizability of those results. Due to the low number of patients with ARDS, we could not group patients as mild, moderate, severe ARDS with regard to the criteria put forth by the Pediatric Acute Lung Injury Consensus Conference (PALICC); thus, subgroup analyses concerning these groups could not be performed. Also, the number of cases with increased physiologic dead space (Vd/Vt ≥ 0.4) was low, leading to a lack of further subgroup analysis.

## Conclusion

It was found that the novel microstream capnometer has no superiority to the traditional mainstream method. Although the mainstream and microstream capnometer measurements had similar correlation values with ABG results, the agreement level of the mainstream method was higher. Although the absolute gradient between both PetCO_2_ methods and PaCO_2_ results demonstrated a consistent increase in the presence of severe pulmonary disease and increased dead space ventilation, both methods showed significant correlations with PaCO_2_ values. Therefore, in the presence of severe pulmonary disease and/or increased dead space ventilation, it is possible that both PetCO_2_ monitoring methods may be helpful in predicting the trend of PaCO_2_ despite limitations in accuracy.

## Abbreviations

CO_2_: Carbon dioxide
ABG: Arterial blood gas
PICU: Pediatric intensive care unit
MAP: Mean airway pressure
OI: Oxygenation index
SD: Standard deviation
IQR: Interquartile range
PASS: Power analysis sample size
